# Thyrotoxicosis Mimicking ST Elevation Myocardial Infarction

**DOI:** 10.7759/cureus.1323

**Published:** 2017-06-07

**Authors:** Ingrid Rymer De Marchena, Anna Gutman, Julie Zaidan, Harout Yacoub, Wissam Hoyek

**Affiliations:** 1 Internal Medicine, Staten Island University Hospital, Northwell Health; 2 Cardiology, Staten Island University Hospital, Northwell Health

**Keywords:** myocardial infarction, st elevation, coronary angiography, hyperthyroidism

## Abstract

Hyperthyroidism is well known to be associated with cardiac disease. Delay in making the diagnosis and occurrence of complications are common and are associated with a worse outcome.

A 54-year-old male, non-smoker, with no past medical history and no significant family history presented to our hospital with severe left sided chest pain, “crushing” in nature. Electrocardiogram showed ST-segment elevations in the inferior leads. Troponin I level was 0.32 ng/mL (normal range 0-0.05 ng/mL) on presentation. The patient underwent an emergent coronary angiography which showed no evidence of occlusive coronary artery disease. The patient’s symptoms and signs prompted a high suspicion of thyrotoxicosis which was subsequently confirmed by a low thyroid stimulating hormone and high free thyroxine levels. The patient was given Methimazole and atenolol and his symptoms resolved.

Awareness of coronary vasospasm due to thyrotoxicosis should be raised in patients presenting with typical angina pectoris with subsequent normal coronary angiographic results. History and physical examination may suggest underlying hyperthyroidism, but the absence of typical findings does not rule out the diagnosis.

## Introduction

Hyperthyroidism is well known to be associated with heart disease [1]. Although this association was reported in the medical literature since the 20th century, there are frequent delays in making the diagnosis [2]. Cardiac manifestations can occur in the absence of symptoms of hyperthyroidism which can make the diagnosis ever more challenging [2]. We are reporting a case of a middle-aged male presenting with an acute myocardial infarction (MI) secondary to thyrotoxicosis. Heightened awareness of this condition is necessary to prevent adverse outcomes. Informed consent statement was obtained for this study.

## Case presentation

A 54-year-old Middle Eastern male presented to our facility complaining of acute left-sided chest pain that started at rest and remained constant for two hours. The pain was severe, pressure-like, non-radiating and was associated with palpitations, lightheadedness, and diaphoresis. Past medical history was unremarkable and the patient was not taking any medications. He had no history of tobacco, illicit drug or alcohol use and he denied having a family history of cardiac disease. Physical examination showed an anxious male with a resting tremor and mild exophthalmos, with a normal body temperature, heart rate of 92 beats per minute, respiratory rate of 16 breaths per minute, oxygen saturation 99% on ambient air and a blood pressure of 150/79 mmHg.

**Figure 1:**
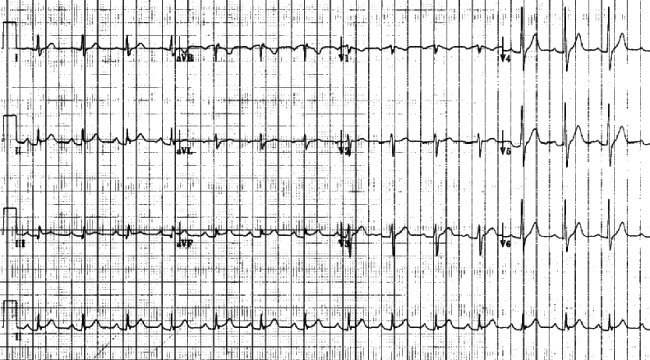
Electrocardiogram showing ST segment elevation in leads II, III and a ventricular fibrillation (VF)

**Figure 2:**
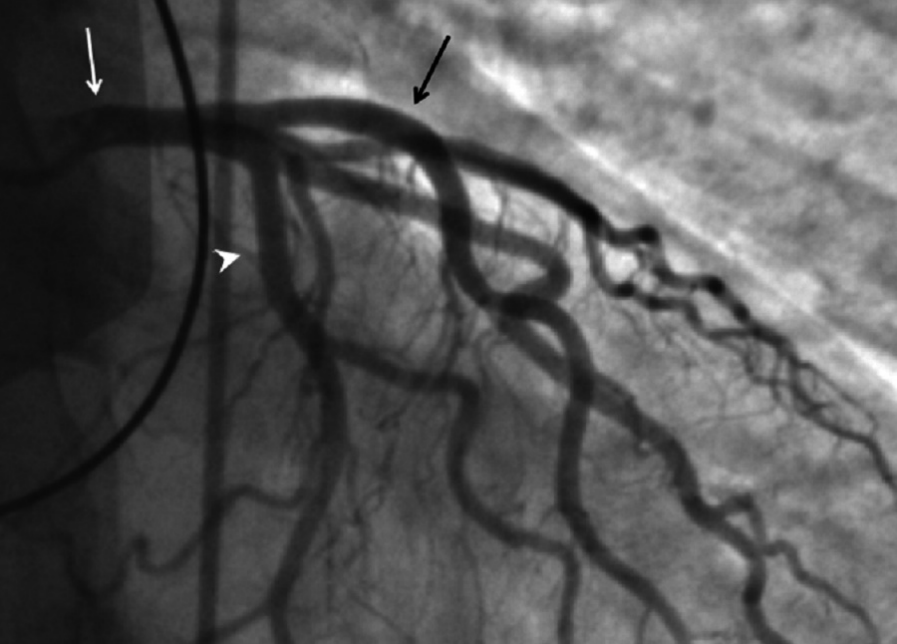
Coronary angiography showing no obstruction White arrow points to the left main coronary artery (CA). Black arrow points to the left anterior descending CA. Arrowhead points to the circumflex branch of the left CA

**Figure 3:**
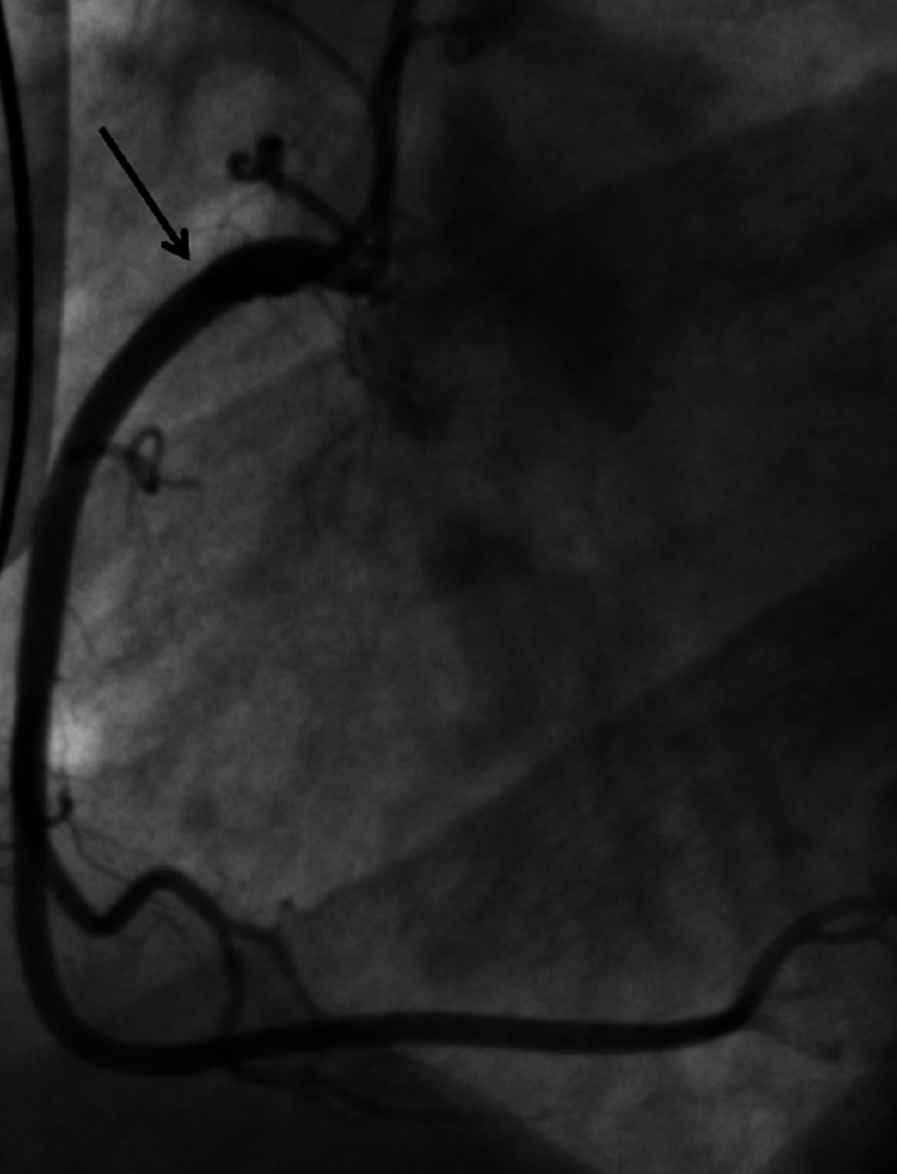
Coronary angiography showing no obstruction Arrow points to the right coronary artery

Electrocardiogram (ECG) showed ST-segment elevation in leads II, III and a ventricular fibrillation (VF) (Figure 1). Initial troponin I level was 0.32 ng/mL (normal range 0-0.05 ng/mL), which increased to 2.2 ng/mL within six hours. The patient underwent an emergency coronary angiography which showed no evidence of occlusive coronary artery disease (Figure 2-3). A chest X-ray and a transthoracic echocardiogram were unremarkable. Serum and urine drug screens were negative and the patient did not have any electrolyte abnormalities. Given the patient’s symptoms of palpitations, diaphoresis, tremors and the evident exophthalmos, thyrotoxicosis was suspected. A thyroid panel showed a thyroid-stimulating hormone of < 0.01 IU/ml (normal range 0.27-4.2 IU/mL) and free Thyroxine levels > 7.8 ng/dl (normal range 0.9-1.8 ng/dL), which suggested a diagnosis of hyperthyroidism. Methimazole and atenolol were started and his symptoms resolved within two weeks. Further investigations showed elevated thyrotropin receptor antibodies confirming the diagnosis of Grave’s disease.

## Discussion

The association between hyperthyroidism and cardiac diseases was first described in the mid 20th century [3]. Coronary events occur 2.6 times more frequently in the presence of high free triiodothyronine levels as compared to normal levels [4]. Reported cardiac events in this setting include myocardial ischemia, angina, arrhythmia, congestive heart failure and sudden death [1, 5]. Proposed hypotheses consist of significant underlying atherosclerosis of the coronaries, overactive sympathetic system, coronary embolization, direct damage to the coronaries and disruption of the blood supply to oxygen demand [1, 6].

In a 2015 review article, it was found that six out of 21 patients presenting with thyrotoxicosis related acute MI had symptoms related to hyperthyroidism. Out of the 21 patients, normal coronary angiography was noted in 13 patients and coronary vasospasm without thrombus occlusion was found in three patients. Symptoms were self-limiting in 18 out of the 21 patients, one had recurrent angina and two died due to their disease. Out of the 21 patients, eight were diagnosed with Graves’ disease, three had painless thyroiditis, two had iatrogenic thyroiditis, one had post-surgical thyrotoxicosis and six remained undiagnosed [7].

Vasospastic angina, previously known as Prinzmetal angina is secondary to a transient coronary vasospasm which occurs in up to 20% of hyperthyroid patients, yet is difficult to confirm [8]. Clinically, thyrotoxicosis-related coronary vasospasm presents as non-exertional chest pain that increases rapidly over time and stops suddenly with control of the thyrotoxicosis [6, 9]. Diagnosis is suggested by finding a reversible coronary artery stenosis on coronary angiography. However, previous reports do not support the use of coronary angiography as a first diagnostic test in patients presenting with chest pain with underlying uncontrolled hyperthyroidism because iodine containing agents (e.g. those used in coronary angiography) have the potential to induce thyrotoxicosis [1, 5]. Thyrotoxicosis induced vasospasm can occur at any time of day which differentiates it from variant angina-related vasospasm that typically occurs in the morning [6]. Possible mechanisms of thyrotoxicosis-induced vasospasm include enhanced coronary sensitivity to vasoconstrictors and reduced sensitivity to vasodilators [1]. Furthermore, coronary vasospasm can promote atherosclerosis by accelerating the formation of a thrombus and delaying fibrinolysis [1]. Treatment of thyrotoxicosis-related coronary vasospasm consists of anti-thyroid and anti-anginal medications [1]. Data supports the use of nitrates and calcium channel blockers over beta-blockers due to the possibility of alpha-adrenergic stimulation and subsequent enhanced coronary vasoconstriction [10]. 

In our case, the patient presented with ST segment elevations in leads II, III and a VF, pressure-like chest pain and an elevated troponin level. The presentation suggested an acute myocardial infarction (MI) despite the apparent lack of significant cardiac risk factors. An emergency coronary angiography did not show occlusive disease of the coronary arteries. Thyroid function tests showed elevated levels of thyroid hormones. In the setting of the above laboratory and angiographic findings, the patient’s clinical presentation was attributed to a coronary vasospasm as a result of thyrotoxicosis.

## Conclusions

Coronary vasospasm due to thyrotoxicosis should be suspected in patients presenting with typical angina with normal coronary angiography results. History and physical examination may suggest underlying hyperthyroidism, but the absence of typical findings does not rule out the diagnosis. Enhanced awareness of this entity is required in view of its potential devastating and even fatal complications.

## References

[1] Kuang XH, Zhang SY (2011). Hyperthyroidism-associated coronary spasm: A case of non-ST segment elevation myocardial infarction with thyrotoxicosis. J Geriatr Cardiol.

[2] Al Jaber J, Haque S, Noor H (2010). Thyrotoxicosis and coronary artery spasm: Case report and review of the literature. Angiology.

[3] Jeffers WA, Littman DS, Rose E. (2017). The infrequency of myocardial infarction in patients with thyrotoxicosis. Am J Med Sci.

[4] Zhou D, Qu Z, Wang H (2015). Severe hyperthyroidism presenting with acute st segment elevation myocardial infarction. Case Rep Cardiol.

[5] Common K, Milburn KS, Cawood TS (2017). Coronary artery spasm due to thyrotoxicosis. N Z Med J.

[6] Canpolat U, Sunman H, Gurses KM (2012). Vasospastic angina in a patient with hyperthyroidism. Herz.

[7] Zheng W, Zhang YJ, Li SY (2015). Painless thyroiditis-induced acute myocardial infarction with normal coronary arteries. Am J Emerg Med.

[8] Dedov II, Kalashnikov Vlu, Terekhin SA (2017). Fatal coronary artery spasm in a patient with thyrotoxicosis. Kardiologiia.

[9] Somerville W, Levine SA (1950). Angina pectoris and thyrotoxicosis. Heart.

[10] Kim HJ, Jung TS, Hahm JR (2011). Thyrotoxicosis-induced acute myocardial infarction due to painless thyroiditis. Thyroid.

